# On the Significance of Microtubule Flexural Behavior in Cytoskeletal Mechanics

**DOI:** 10.1371/journal.pone.0025627

**Published:** 2011-10-05

**Authors:** Mehrdad Mehrbod, Mohammad R. K. Mofrad

**Affiliations:** Molecular Cell Biomechanics Laboratory, Department of Bioengineering, University of California, Berkeley, California, United States of America; University of Manchester, United Kingdom

## Abstract

Quantitative description of cell mechanics has challenged biological scientists for the past two decades. Various structural models have been attempted to analyze the structure of the cytoskeleton. One important aspect that has been largely ignored in all these modeling approaches is related to the flexural and buckling behavior of microtubular filaments. The objective of this paper is to explore the influence of this flexural and buckling behavior in cytoskeletal mechanics.

*In vitro* the microtubules are observed to buckle in the first mode, reminiscent of a free, simply-supported beam. *In vivo* images of microtubules, however, indicate that the buckling mostly occurs in higher modes. This buckling mode switch takes place mostly because of the lateral support of microtubules via their connections to actin and intermediate filaments. These lateral loads are exerted throughout the microtubule length and yield a considerable bending behavior that, unless properly accounted for, would produce erroneous results in the modeling and analysis of the cytoskeletal mechanics.

One of the promising attempts towards mechanical modeling of the cytoskeleton is the tensegrity model, which simplifies the complex network of cytoskeletal filaments into a combination merely of tension-bearing actin filaments and compression-bearing microtubules. Interestingly, this discrete model can qualitatively explain many experimental observations in cell mechanics. However, evidence suggests that the simplicity of this model may undermine the accuracy of its predictions, given the model's underlying assumption that “every single member bears solely either tensile or compressive behavior,” i.e. neglecting the flexural behavior of the microtubule filaments. We invoke an anisotropic continuum model for microtubules and compare the bending energy stored in a single microtubule with its axial strain energy at the verge of buckling. Our results suggest that the bending energy can exceed the axial energy of microtubules by 40 folds. A modification to tensegrity model is, therefore, proved necessary in order to take into account the flexural response of microtubules. The concept of “bendo-tensegrity” is proposed as a modification to contemporary cytoskeletal tensegrity models.

## Introduction

**Figure 1:**
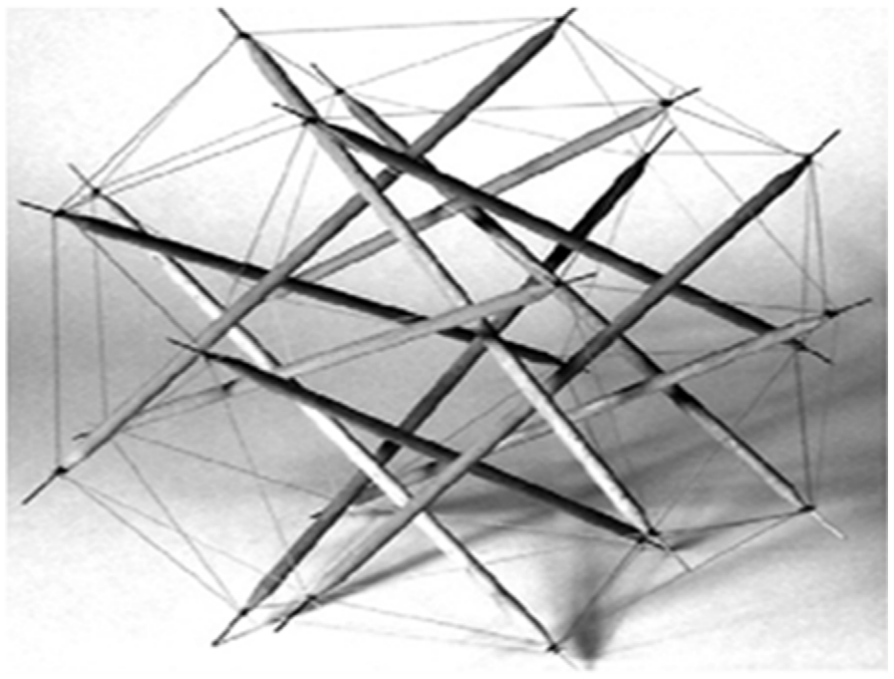
A typical tensegrity model used to simulate the cell cytoskeleton [**8**].

Living cells actively respond to their mechanical environment, altering their proliferation rate, cytoskeletal configuration, and gene expression pattern when exposed to a mechanical perturbation. The details of how cells sense mechanical signals and how mechanical signals are transduced and transmitted from the extracellular matrix (ECM) throughout the cell have remained ambiguous [1], [2]. This has motivated quantitative models for the cytoskeleton [1], as a mechanical structure hosting and participating in signaling pathways of the cell [1], [2], [3]. Among these models, the cytoskeletal tensegrity model (see Fig. 1) has received traction in elucidating several aspects of the cellular response to mechanical stimuli. The model describes the cytoskeletal filaments as discrete members that come together to form a discontinuous, so-called *tensegrity* (tension integrity) structure [4], [5], [6], [7]. A tensegrity structure, by definition, must fulfill an important criterion: It should be composed of only tensile and compressive members, meaning that each and every member must bear either pure tension or compression. Furthermore, the tensegrity structure should be free from any shear-introducing structural behavior (i.e. bending and torsion) [8]. Such a condition would imply that all members must be straight, all joints must be moment-free (or hinge), and finally, loads should be applied exclusively to joints [9]. Additionally, a tensegrity structure owes its stability to the pre-existing tensile stress (pre-stress) in its members, since its slender members could transfer the load only when they are under tension [5].

**Figure 2:**
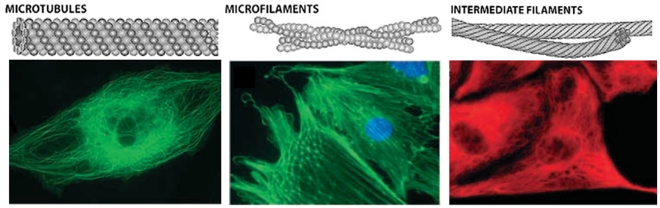
Network of cytoskeletal filaments [**5]**, [8****].

In most eukaryotic cells, the cytoskeleton comprises three types of filaments: actins, microtubules, and intermediate filaments (see Fig. 2) [10]. Experimental studies have shown that actins and intermediate filaments can sustain only tension because of their small cross-sections, whereas microtubules are mostly subjected to compression. Furthermore, the presence of pre-stress in the cytoskeleton has been indicated in several studies [5], [6], [11], [12]. Compression and buckling of microtubules in adherent cells counterbalance some 5% to 30% of the tensile pre-stress [5], [6], while the rest is supposedly counteracted by the ECM [1].

Given its simple incorporation of the structural pre-stress as well as tensile and compressive behavior of the load-bearing members, the tensegrity model provides a robust tool for cytoskeletal modeling and analysis. Nevertheless, this approach has its drawbacks, especially when it comes to the mechanics of microtubules. In this paper, we explore the consistency of the tensegrity hypothesis with the experimental observations on mechanical behavior of microtubules as compressive members of the cytoskeleton, and motivate a modification to this model.

## Methods

Unlike other cytoskeletal filaments, such as actins and intermediate filaments that work together as a network, microtubule filaments typically respond to mechanical excitations as individual structural elements [13]. Molecular structure of microtubules differs from typical polymers as they have considerably larger persistence lengths. From this viewpoint, microtubules behave more like rigid bars. As one of the most rigid cytoskeletal components, microtubules provide support for the cell to maintain its shape [13], [14], [15]. Rigidity of the microtubule filament results largely from its hollow cylindrical shape, composed of α-β tubulin heterodimers that form protofilaments [16]. The microtubule structure is composed of 13 parallel protofilaments oriented longitudinally [17]. Microtubules play critical roles in cell motility, growth, mitosis, and meiosis [10], and act as tracks for motor proteins to carry cargoes across the cytoplasm. The rapid polymerization and depolymerization of microtubules give rise to formation of highly dynamic structures [18].

**Figure 3:**
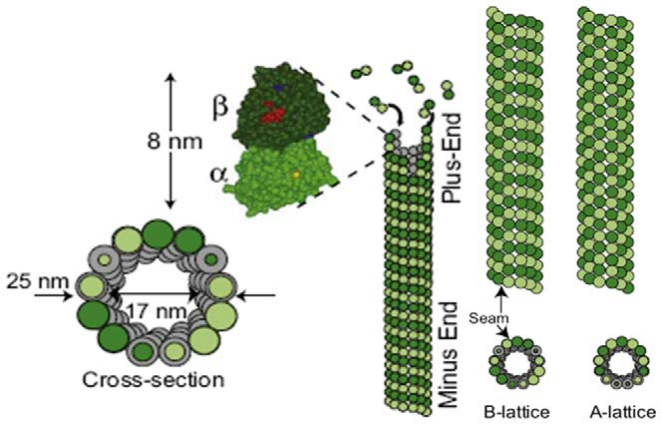
Geometry of a microtubule [**13**].

**Figure 4:**
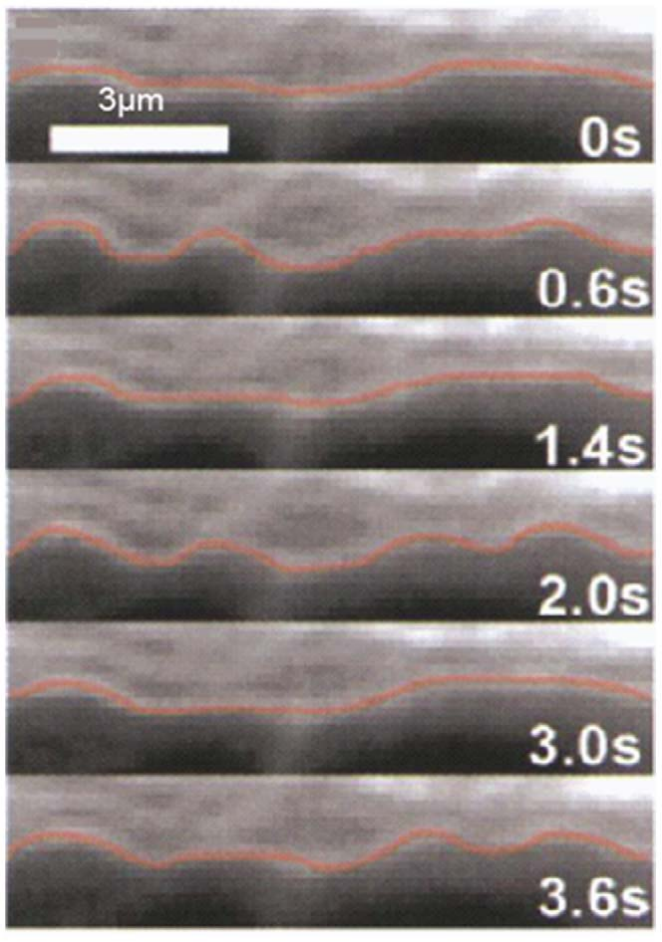
Buckling pattern of a single microtubule during contractile beating of heart cells [**36**].

**Figure 5:**
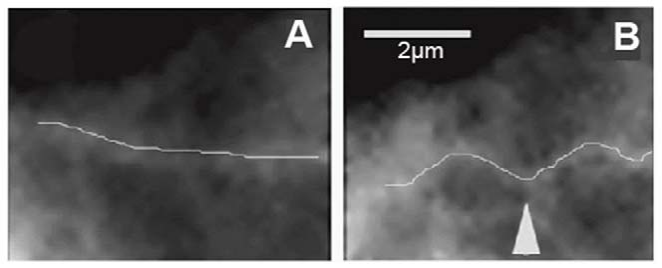
Buckling of a fluorescently-labeled microtubule in living endothelial cells following cell contraction induced by thrombin. (A) The microtubule is fairly straight before the application of the load, and (B) it buckles in small wavelengths when it is loaded. The scale bar is 2 µm [1].

The outer and inner diameters of microtubules are 25 nm and 17 nm, respectively (see Fig. 3). The length of microtubule filaments varies from tens of nanometers to hundreds of micrometers [10], yet at least an order of magnitude less than their persistence length, which is reported to be 0.2–9 mm [18]. However, previous experimental studies have shown that even though the persistence length of microtubule filaments is far greater than their lengths, microtubules do not necessarily appear straight in the cytoskeleton; rather they exhibit periodic curves (see Fig. 4 and 5), suggesting that microtubule filaments sustain compressive forces and hence buckle under compression [5], [6], [11]. As depicted in Fig. 3, microtubules polymerize in two different ways, shaping two lattice forms named as A-lattice and B-lattice, with distinct flexural rigidities. Cytoskeletal microtubules are mostly of the A-lattice type while B-lattice type mostly forms when spontaneous polymerization occurs *in vitro* [18].

**Figure 6:**
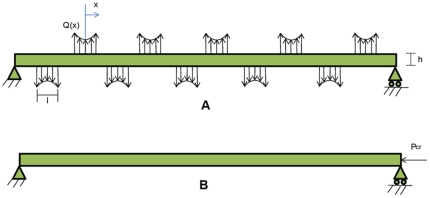
Mechanical models used to estimate the axial and bending energies of the microtubule. (A) A microtubule is modeled as a simply supported beam, being supported by an average number of nine intermediate filaments in constant intervals and each having opposite directions to its neighbors. Load distribution functions over the connection lengths (*Q(x)*) is derived in the text. The connection length is denoted by *l* and the beam depth by *h*. (B) To estimate the axial energy the microtubule model is considered to be under a uniform compressive load. This load equals the critical buckling load of the microtubule when the beam is hinged at its two ends.

Many researchers have attempted to measure the buckling load of single microtubules [11], [17], [19]. Interestingly, the critical buckling load of microtubules in living cells is two orders of magnitude larger than what microtubule filaments show *in vitro* [17]. In addition, individual microtubule filaments buckle in the first mode *in vitro*, while they are often observed to buckle in short wavelengths in living cells (see Fig. 4). The critical axial load of a simply-supported beam composed of an isotropic linear elastic material can be calculated as:
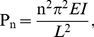
(2)where *n*, *E*, *I* and *L* are the microtubule's buckling mode number, elastic modulus, cross-section's second moment of inertia, and contour length, respectively [20]. *P_n_* is called the *n* ^th^ Euler load, or the buckling mode number, of the beam. For a beam with a rectangular cross-section and transverse isotropic material this relationship should be modified as:
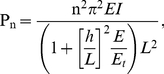
(3)where *h*, *E*, and *E_t_* are the beam depth, longitudinal Young's modulus, and longitudinal-transverse shear modulus, respectively (see Fig. 6) [21]. Note that Eq.3 simplifies to Eq.2 at small *h*/*L* ratios, and takes the same form for cylindrical and rectangular cross-sections. In this study we model a single microtubule filament as a transverse isotropic, hollow beam as suggested by the latest studies [22], [23].

A series of analyses were conducted using a commercially available finite element software package ADINA 8.6 (ADINA R&D, Watertown, MA), aimed at estimating the bending strain energy induced by the lateral linkers and comparing it with the axial strain energy caused by axial forces applied at ends of a single microtubule. Towards this goal, the microtubule filament is simulated by a continuum model of a simply-supported beam. To model a typical microtubule of 13 protofilaments with 3 starts, a one-micrometer-long hollow cylinder with corrected inner and outer radii of 9.9 and 11.5 nm, was employed as proposed by Shen [22]. Bonds between tubulin dimers are believed to be significantly stronger along the protofilament's longitudinal direction than those on the microtubule cross-sections perpendicular to its axis [16], justifying a transverse-isotropic material model for microtubules as adopted in this analysis.

Two scenarios are considered: (i) an isolated microtubule filamement subjected to only one critical axial load (i.e. the force that causes the microtubule to buckle); (ii) the identical microtubule filament bearing an axial compressive load as large as its critical buckling load while being supported at constant intervals along its length by identical lateral forces *in vivo* (see Fig. 6). Scenario (i) mimics the case for *in vitro* microtubule buckling tests while case (ii) is a typical condition of the microtubule when embedded in the filamentous milieu of the cell. Experimental data indicate that the critical load in case (ii) is two orders of magnitude larger than that in case (i) [14]. Since all mechanical and geometrical properties of the two microtubule filaments (e.g. their bending stiffness and length) are identical, this discrepancy should stem from different boundary conditions, reflected in buckling mode numbers, *n* (see Eq. 3), that determines the microtubule's buckled shape. Microtubules buckle in mode number one, shaping like a half cycle of a periodic curve, if they are laterally unconstrained. For the higher mode numbers to take place far greater deformation energies are required, thus they occur less likely if the microtubule filament is not restrained laterally. Both cases mentioned are typical idealized loading conditions to evaluate the order of magnitude of flexural energy stored in a microtubule as a result of subsequent linkages to its filamentous surrounding. Although randomness of the connection intervals imposes a high variance to this calculation, we seek for an average value of flexural energy that is sustained by the microtubule in a typical cytoskeletal environment. Importantly, any wave shape formed by an arbitrary loading condition could be decomposed into a combination of different buckling mode numbers.

According to Eq.3, the mode number that yields a critical buckling load of two orders of magnitude greater than the first mode is mode number 10. Practically, however, the tenth mode occurs when there exist nine additional lateral supports. Therefore, it is assumed that there are on average nine linkages between the microtubule and its adjacent filament network. For the sake of simplicity, forces applied by supports are all identical in distribution and each force has opposite direction to its neighbors. This is a typical distribution which would yield a periodic deflection pattern. Additionally, it is speculated that forces are exclusively applied by actin linkages. Linkages between microtubules and actins or intermediate filaments are formed by a series of participating proteins. It is reported that some proteins, including kinesin, myosin, plectin, and MAP2c, are potential linking agents of actins and intermediate filaments to microtubules [24], [25], [26], [27]. Generally, two actin-microtubule cross-linking mechanisms are proposed [25]: regulatory and structural connections. The scope of this paper is limited to structural linkages wherein a physical connection exists between actins and the microtubule. These connections enable actins to support the microtubule when they are under tension [25].

**Figure 7:**
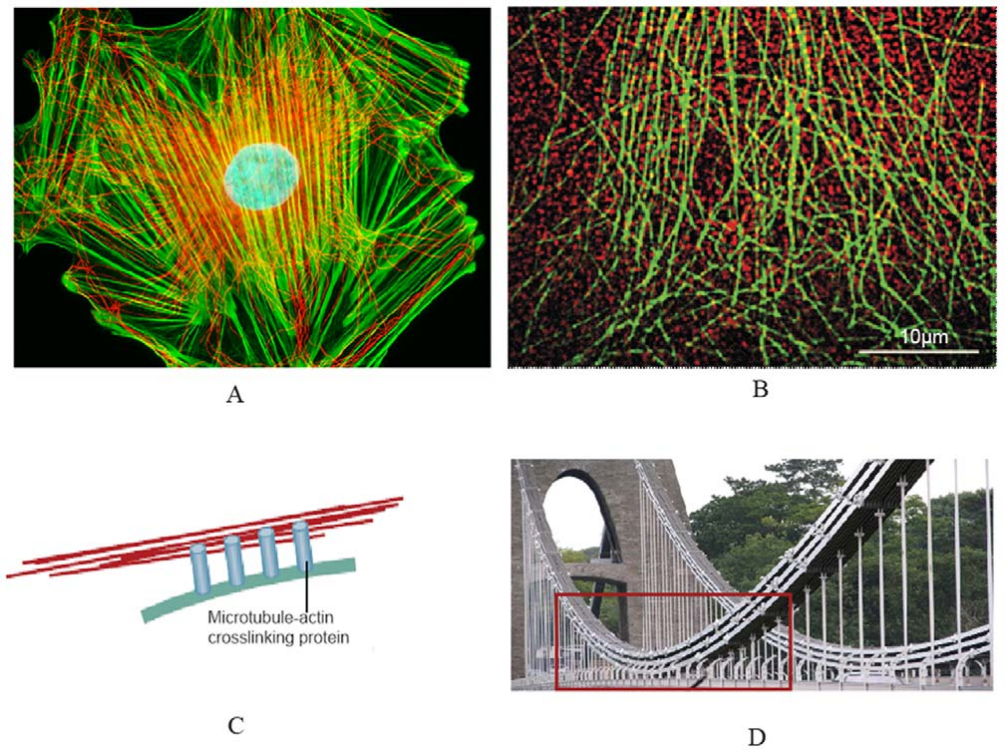
Analogy between a microtubule-actin connection and a suspension bridge. (A) Immunofluorescently labeled rat thoracic aorta cells illustrates actin (red) and microtubule (green) networks. (http://learn.hamamatsu.com/galleries/digitalimages) (B) Connections between actin filaments (red) and microtubules (green) in growth cones from Aplysia bag cell neurons [25]. (C) A schematic view of the microtubule-actin connection via linking proteins [25] (D) In a suspension bridge the main curved cable is attached to the relatively straight deck by several vertical cables. (The analogy area is boxed.) (http://www.travelpod.com/).

Since linkages appear as series along the microtubule length, the overall configuration of an actin-microtubule connection resembles the deck-main cable connection in a suspension bridge (see Fig. 7). In this analogy the microtubule acts as the bridge deck because of its high flexural stiffness that gives rise to its substantially lower curvature relative to the actin filament, which is thought of as the main (curved) cable of the suspension bridge. Therefore, we neglect the microtubule local curvature as compared to the actin curvature at the linking zone. Straight vertical cables, on the other hand, would play the role of the linkage proteins, connecting the microtubule and actin filament. In addition, the entire structure and loads are assumed to remain in a plane.

Lateral supporting of the microtubule filaments originates largely from the neighboring filament network but the surrounding viscous cytosol could also exert lateral forces when the filament moves laterally as it buckles. In addition to the elastic deformation of the surrounding cytoskeletal network, the buckling motion induces a viscous flow in the cytosol [14], [28], [29]. However, the viscous part of the lateral load gradually vanishes and eventually the microtubule filament is left with a lateral support coming solely from the filament network [14], [23], [30]. Hence, the strain energy calculation in our model applies to the case when the microtubule is supported only by its neighboring filaments. It is worth mentioning that a slippery ionic layer around microtubule filaments prevent the shear interaction between microtubules and their mechanical surrounding [23]. Therefore, in our model shear stresses exerted on the microtubule by its surrounding cytosol are neglected.

## Results

To incorporate mechanical effects of other cytoskeletal filaments on microtubules, most researchers have simulated a microtubule filament as a beam surrounded by an elastic continuum [14], [22], [23], [31]. However, the filamentous actin network is far from a continuum, and moreover, the connections between these actin filaments and microtubules are formed in discontinuous, limited intervals. In this study, a semi-discrete method is adopted and it is assumed that loads on the microtubule filament are applied at alternating continuous intervals (see Fig. 6), whereas load distribution pattern for each interval will be derived in the following.

**Figure 8:**
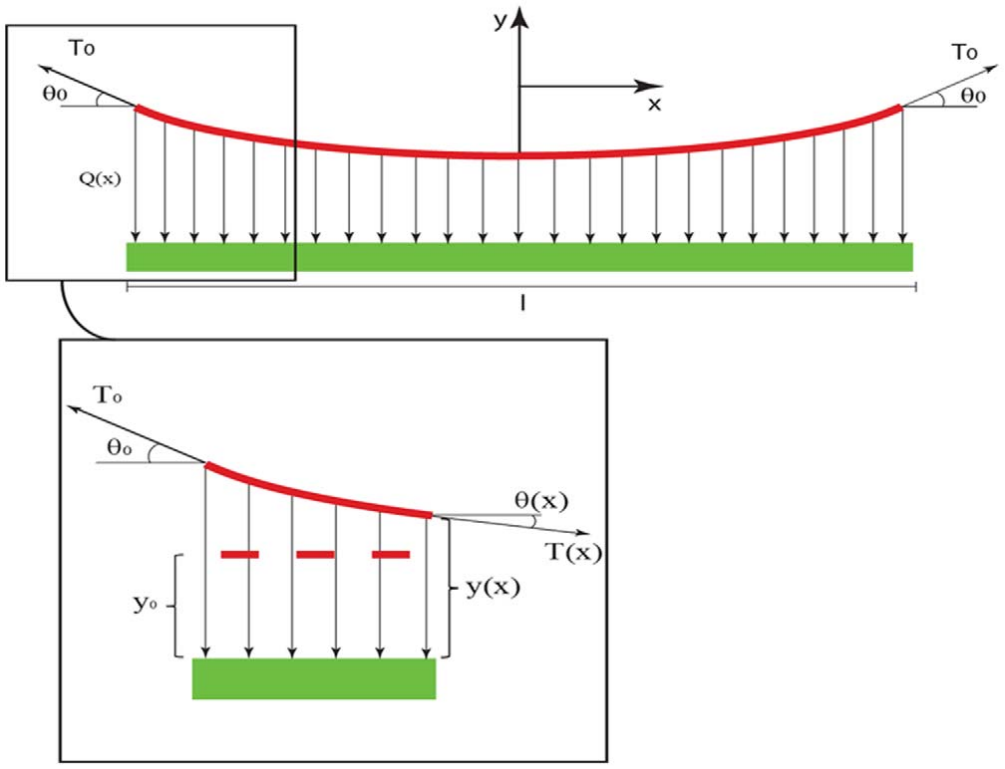
Details of the free body diagram for a microtubule-actin connection length. (Top) The actin filament (red) is connected to the microtubule (green) via linker proteins, which are substituted here by their mechanical effect as vertical forces. *T_0_* is the tensile force sustained by the actin filament, *θ_0_* is called the start angle, and *Q(x)* represents load per unit length (Bottom) a cut-out region of the connection length is illustrated. The red dashed and solid lines represent the actin filament before and after the axial loading. In an unloaded status the actin filament should stay parallel to the microtubule so that no linker protein is mobilized. After the loading, as *T_0_* direction is not necessarily parallel to the microtubule linker proteins take different lengths.

Depicted in Fig. 8 are the connection details of an actin filament (red) and a microtubule (green) by vertical linkages in one connection length as described in our model. Using the force equilibrium and free body diagram sketched in Fig. 8, one can write:
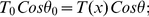
(4.a)

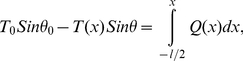
(4.b)where *T_0_* and *T(x)* are the actin axial forces in the beginning of the interval and at the position x from the interval center, where *x* could vary between −l/2 and l/2. Here, *θ_0_* and *θ* are angles between the actin filament tangent and microtubule direction in the beginning of the interval and the position *x*. *Q(x)* is an unknown load distribution function over the interval length. Assuming that the connection proteins act like linear springs with stiffness *k* and with dimensions *dx* and *dz* along the microtubule length and perpendicular to it, respectively, we have:
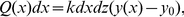
(5)where *y_0_* and *y(x)* are the lengths of a single linker protein at position *x* along the microtubule length before and after the actin filament is loaded. Substituting (5) in (4.b), combining (4.a) and (4.b), and differentiating with respect to *x* yields:
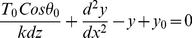
(6)Solving the differential equation results in:
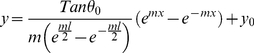
(7)

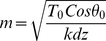
(8)And finally, substituting (7) in (5), we obtain the load distribution as a function of *x*:
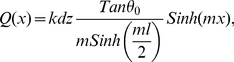
(9)which is the estimated load distribution corresponding to one microtubule-actin connection. Identical load distributions are assumed at the other eight connections (see Fig. 6). This leads to a semi-discrete loading pattern that attempts to mimic the filamentous environment around the microtubule filament. Using concentrated point loads in our continuum model resulted in considerable stress concentrations, which induced mostly localized deflections in the microtubule rather than a harmonic deflection in the microtubule body as a whole; a semi-discrete loading yields a deflection profile reminiscent of physiological observations (see Fig. 5).

**Figure 9:**
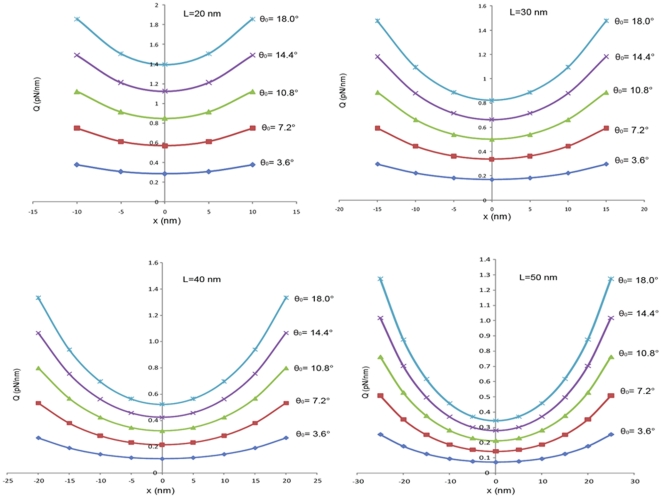
Microtubule-actin connection load distribution is a function of the angle θ_0_ and the connection length.

The axial load sustained by actins (*T_0_*) is assumed to be 50 pN, similar to a typical physiological stretch [32]. The linker protein stiffness (*k*) and cross-sectional dimension (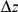) are taken as 0.15 pN/nm and 2 nm based on values reported for myosin [32], [33]. Other model properties are listed in Table 1. For several values of *L* and 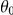, the load distribution function for a single microtubule-actin connection is calculated and presented in Fig. 9. For each value of *L*, the load applied on the microtubule by the actin filament increases monotonically with *θ_0_* (see Eq.9). However, there is an upper limit imposed in practice: linking proteins generally cannot transfer loads more than ∼5 pN per protein [33]. This criterion limits *θ_0_* to small values (less than about 18°).

**Table 1:** Microtubule material and geometric parameters.

Young's Modulus	Poison's Ratio	MT length	MT Inner radius	MT Inner radius
Eyy = Exx = 1 Mpa Ezz = 1 Gpa	ν_xy_ = ν_xy_ = 0.03 νrz = 0.3	1 µm	9.9 nm	11.5 nm

For distributions with higher connection lengths (*l*), *Q(x)* confers lower values as expected. Interestingly, however, larger connection lengths give rise to sharper distribution patterns with smaller minima. If this length is smaller than 20 nm, the single linker load exceeds 5 pN, and if it is larger than 50 nm it induces abnormal deflections in the microtubule. Considering these criteria, the allowable load distributions were determined and applied on the microtubule filament model (Fig. 6.a). The model was analyzed using the finite element method to attain the bending strain energy corresponding to each distribution. Since *in vivo* microtubules are observed to buckle under compressive loads, the analysis of the microtubule model was conducted one more time with a concentrated, compressive load (200 pN) at its ends to compute the axial strain energy for a buckled microtubule (Fig. 6.b).

**Figure 10:**
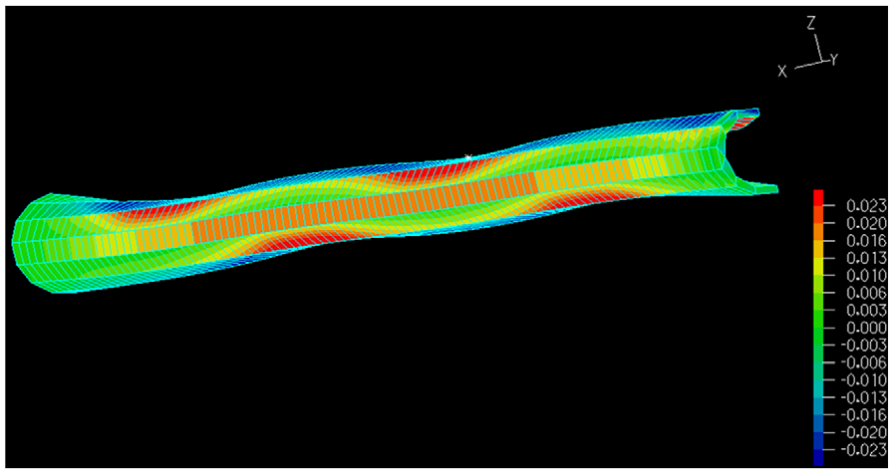
Microtubule deformation under lateral load. Deflection is not to scale; the picture illustrates a microtubule half cross-section with half length. Normal strain parallel to the loading plane and orthogonal to the microtubule axis is illustrated in the picture (*ε_zz_*).

**Figure 11:**
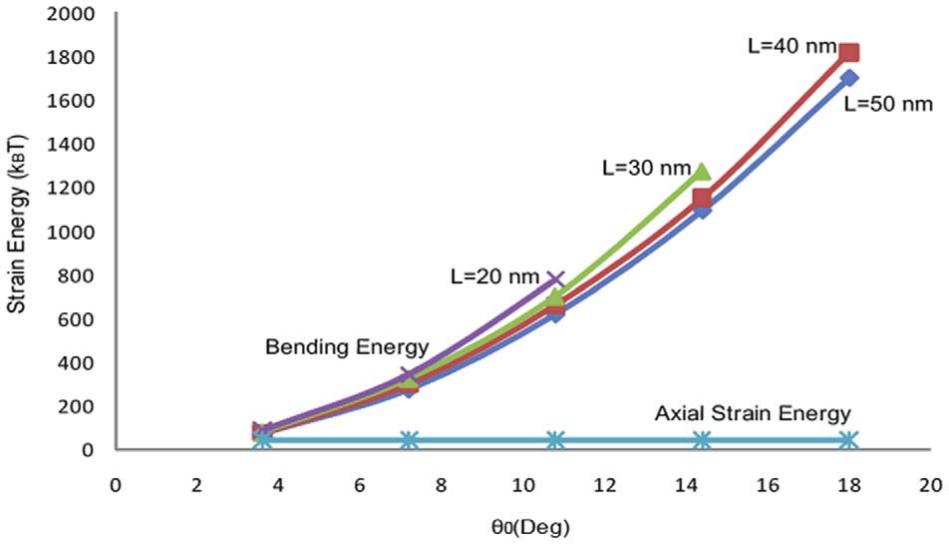
Comparison between microtubule bending strain energy induced by the lateral connection to actins, and the microtubule maximum axial strain energy under critical buckling load.

A microtubule deflection pattern is illustrated in Fig. 10. To reduce the computational cost, symmetry of the microtubule geometry is invoked. Half cross-section of the microtubule with full length was employed to compute the axial energy while the microtubule half cross-section with half length was used to simulate the laterally supported microtubule. Surprisingly, despite the significant differences in the load distributions due to various connection lengths, the bending energy versus 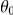 (we call it start angle) distribution curves fall in a narrow band (see Fig. 11).

The axial energy corresponding to the critical load remains unchanged as the connection length increases and it is almost equal to the bending energy at small start angles (less than 4°). Nevertheless, the bending energy increases exponentially as the start angle is increased until it reaches up to 40 times the axial energy stored in the microtubule filament. This implies that the cytoskeletal structure under normal physiological loads stores a considerable amount of flexural energy, at least in microtubules. This observation and the notion that the bending energy is induced by the connection of microtubules to actin filaments, which are tensile cytoskeletal members, leaves us with a structure whose tensile members are jointed to its compressive members not necessarily at the end points. These tensile members apply an ample amount of bending to microtubules and this violates one of the basic tensegrity assumptions.

## Discussion

The tensegrity model for the cytoskeleton has received considerable traction in the cell mechanobiology literature. It models the cytoskeleton as a combination of several struts and cables [1], [2], [5], [6], [8], [11], [34], [35]. The cellular tensegrity model successfully explains several observations in mechanics of the cell, including the cytoskeletal pre-stress, discrete nature of the cytoskeletal network, and the action-in-distance phenomenon [4], [5], [6]. Furthermore, simulation of the cell based on the tensegrity assumption is both easy and computationally efficient. Nonetheless, the tensegrity model comes short of taking into account the flexural response of microtubules, which introduces a significant error in the mechanical analysis of the cytoskeleton. Theoretically, an axially loaded beam could reach equilibrium in any arbitrary mode. However, beyond the first critical load the beam assumes an unstable equilibrium in which any subtle change in its shape leads to destabilization. It is therefore unlikely that a free microtubule filament reaches higher buckling modes while being bombarded by the surrounding Brownian forces. As a result, the high critical load and buckling strength of microtubules *in vivo* should be attributed to some kind of lateral bracing of microtubules in the cell milieu. In fact, microtubules in the living cell environment are surrounded by an interconnected network of actins and intermediate filaments. Some researchers suggest these connections may provide lateral support for microtubules, which could be sufficient for preventing them from buckling in the first mode [6], [36], [37]. Because the contour length and bending rigidity (*EI*) of microtubules studied are identical in isolated (free) microtubules *in vitro* and in living cells, the difference in the critical loads could only pertain to their buckling mode numbers. The unsupported microtubule simply buckles in the first mode, but the microtubule constrained by its surrounding buckles in higher modes.

Conducting a mechanical analysis on a single microtubule connected to actin filaments at nine intervals, a physiologically probable load distribution was represented here to the microtubule. It was shown that when the cytoskeleton is loaded, which is always the case in adherent cells, the microtubule filament's high bending capacity is mobilized due to the stretch of actins and intermediate filaments connected to microtubules. Actin filaments reportedly sustain up to 110 pN *in vivo* [32] and their binding to microtubules induces a considerable flexural behavior in microtubules. Actin and intermediate filaments are mostly crosslinked to each other somewhere in the middle of their lengths and not necessarily at their ends. This does not cause a problem in our tensegrity modeling of actins and intermediate filaments as we can still assume the free lengths of actin and intermediate filaments between two connections each as a tensegrity member (hinge-ended). This, however, is not applicable to microtubules because of their strong cross-section and high moment-bearing capacity. If we assume the microtubule's free length between two actin connections as a tensegrity member, this member is no longer hinged to its neighboring members. The underlying principle here states that the large bending rigidity of microtubules do not allow us to neglect their flexural behavior when they are subjected to lateral forces. The ratio of the characteristic bending energy of the filament to the characteristic thermal energy of the environment (Eq.10) determines the significance of the bending behavior induced by the thermal environment [38]:
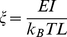
(10)The value of *ξ* for microtubules is always larger than 200, which indicates that the mechanical effect of the thermal environment is negligible and microtubules could be modeled reasonably with elastic rods rather than flexible chains.

From a nanoscale perspective, material properties of microtubule filaments are size-dependent, which calls for an adjustment in microtubule continuum models, namely the “non-local continuum theory” [19]. Yet, the continuum theory is sufficient for the purpose of our analysis, firstly because in the physiological temperature (37°C) and for high length to characteristic radius ratios (L/R>100), as assumed in this study, non-local effects are fairly small (around 10%) and length-independent [16], [17], [19]. Additionally, the primary goal of this paper is to conduct a scaling analysis and compare the scales of bending and axial strains built up in a microtubule filament, rather than performing a detailed analysis of the microtubule. Treating microtubules as isotropic solids, rather than transverse-isotropic continua, has been another source of variation in flexural rigidity calculations based on experimental values of persistence length in many of previous studies [16], [17].

The current tesegrity-based models largely neglect the support that actins and intermediate filaments provide for microtubules, which on average produces a 100-fold increase in the sustainable axial load borne by microtubules. In addition, our analysis indicates that the bending strain energy stored in a microtubule when the actin filaments are under tension could exceed the axial energy caused by the compressive force, by at least an order of magnitude. Such domination of the flexural behavior in microtubules clearly violates the tensegrity presumption. A meaningful amount of the external work done on the cell is spent to bend microtubules. In case the flexural behavior is neglected, the analysis would mistakenly redistribute this extra energy as an additional axial energy between the members, conferring wrong amounts of axial forces and displacements for the elements and nodes.

In order to address this drawback, one can envision a “bendo-tensegrity” model for a more accurate representation of microtubule's role in cell mechanics. Similar to the tensegrity model, in a bendo-tensegrity model the actin and intermediate filaments solely bear tension, but the flexural response of microtubule filaments as well as their compressive action are taken into account by connecting nine tensile filaments at even intervals along the length of each microtubule. Therefore, while preserving the discrete nature of the model and its simplicity to a great extent, significantly more accurate predictions of the cell deformation pattern and force distribution among the cytoskeletal members could be achieved. Cytoskeletal networks mapped by imunofluorescence imaging can supply the bendo-tensegrity model with a temporal configuration and the output would be the temporal distribution of forces among the cytoskeletal members. Estimating the force in cytoskeletal filaments is essential to cell behavior studies specifically cell migration and focal complex formation.

This study demonstrates that an accurate perspective of the cytoskeleton is only achievable if we employ microtubule filaments' bending capacity. This can be accomplished by mounting microtubules with additional cables attached to them to represent the crucial role of the intermediate or actin filaments. Finally, coupling of computational works employing the bendo-tensegrity model with experimental cell mechanics studies will open up a new area of cell mechanics modeling that could be a promising subject for further research.
